# Removal of toluene as a toxic VOC from methane gas using a non-thermal plasma dielectric barrier discharge reactor

**DOI:** 10.1039/d1ra04772h

**Published:** 2021-08-12

**Authors:** Faisal Saleem, Abdul Rehman, Farhan Ahmad, Asif Hussain Khoja, Farhan Javed, Kui Zhang, Adam Harvey

**Affiliations:** School of Engineering, Newcastle University Newcastle upon Tyne NE1 7RU UK h.faisalsaleem@gmail.com; Department of Chemical and Polymer Engineering, University of Engineering and Technology Faisalabad Campus Lahore Pakistan; Department of Chemical Engineering, University of Engineering and Technology Lahore Pakistan; Fossil Fuels Laboratory, Department of Thermal Energy Engineering, U.S.-Pakistan Centre for Advanced Studies in Energy (USPCAS-E), National University of Sciences & Technology (NUST) Sector H-12 Islamabad 44000 Pakistan

## Abstract

Methane is the main component of biogas, which could be used as a renewable energy source for electricity, source of heat, and biofuel production after upgrading from biogas. It also contains toxic compounds which cause environmental and human health problems. Therefore, in this work, the removal of a toxic compound (toluene) from methane gas was studied using a dielectric barrier discharge (DBD) reactor. It was observed that the removal of the toxic compound could be achieved from methane carrier gas using a dielectric barrier discharge reactor, and it depends on plasma input power. The maximum removal of the toxic compound was 85.9% at 40 W and 2.86 s. The major gaseous products were H_2_ and lower hydrocarbons (LHC) and the yield of these products also increases with input power. In the current study, the yield of gaseous products depends on the decomposition of toxic compounds and methane, because the decomposition of methane also produces H_2_ and lower hydrocarbons. The percentage yield of H_2_ increases from 0.43–4.74%. Similarly, the yield of LHC increases from 0.56–7.54% under the same reaction conditions. Hence, input power promoted the decomposition of the toxic compound and enhanced the yield of gaseous products.

## Abstract 2

The removal of toluene as a toxic volatile organic compound from methane gas, which is the major component of biogas, is carried out using a non-thermal plasma dielectric barrier discharge reactor.

## Graphical abstract

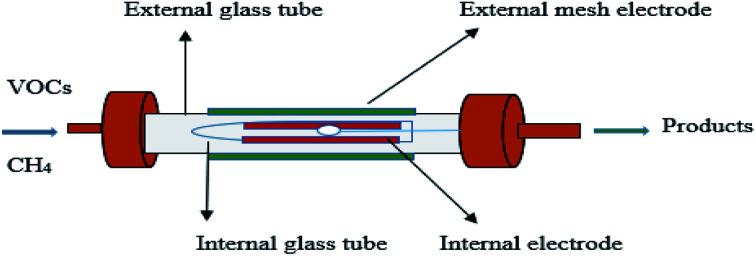

## 1. Introduction

In recent years, non-thermal plasma (NTP) has received significant attention in gas cleaning and removal of VOCs due to its simple operation and the ability to handle high and low concentrations of VOCs.^1–11^ NTP is considered as non-equilibrium plasma due to a very high-temperature difference between electrons and heavy species. Electrons are produced and accelerated by the electric field, which collide with the molecules and atoms passing through the plasma reactor. Some electrons have sufficient energy to ionize these gas atoms or molecules upon collision. The energy required to dissociate/ionize the background gases is in the range of 5–25 eV.^12^ It was observed that a sufficient number of high energy electrons are present in a Maxwellian distribution to dissociate/ionize background gas molecules.^13^ The molecules, ions, and gas atoms remain at modest temperature, whereas electrons in comparison are very hot. It was reported that the input power absorbed by the electrons was very high (*i.e.* 10^2^–10^3^ times) compared to the heavy particles.^14^ However, the energy transferred from electrons to heavy particles is small due to the lower mass of the electrons.^15^

The treatment of pollutants *via* NTP is mostly carried using a dielectric barrier discharge (DBD) due to its ease of operation and the availability of an efficient and economical power supply.^16–23^ Due to the combined effect of non-equilibrium plasma properties and ease of operation at ambient pressure, DBD reactors are being used for industrial applications.^24,25^ The ability of the DBD reactor to simple scaling up is also a well-known feature for the commercial applicability of this process.^26,27^ Moreover, cost-effective and reliable power supplies are also easily available to generate NTP in DBD reactors. The frequency range (1 kHz to 10 MHz) and the pressure range (10–500 kPa) are normally preferred for industrial applications of the DBD reactor.^28^ Currently, DBD reactors are widely used for a range of industrial applications such as the treatment of pollutants causing anthropogenic emissions into the atmosphere, ozone generation, surface treatment, ultraviolet lamps, mercury-free fluorescent lamps, and large-area flat plasma displays.^28,29^

Recently, many researchers focused on the removal of VOCs using air as a carrier gas.^30–34^ However, in biogas, the major component of gas is CH_4_, which could be up to 75% depending on the type of feedstock and operating condition of the digester.^35^ To the best of our knowledge, the removal of the toxic compound using CH_4_ as a carrier gas in a DBD reactor has not been studied so far. Therefore, it was very important to investigate the removal of toluene using CH_4_ as a carrier gas to evaluate the performance of NTP. Herein, toluene was chosen as a model compound due to its presence in abundance in biogas. Toluene is also used in many industrial processes.^36^ The concentration of VOCs was kept higher (33 g Nm^−3^) than the amount of VOCs present in biogas, leaving operational margin for scaling up into industrial practice.^37^

## 2. Experimental setup

**Fig. 1:**
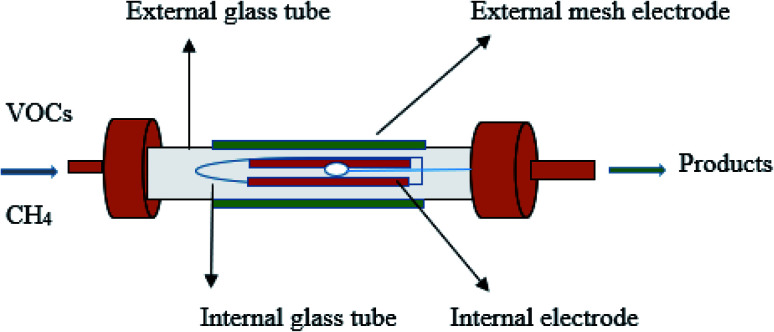
Schematic diagram of the DBD reactor.

Fig. 1 shows the schematic diagram of the DBD reactor. The NTP coaxial DBD reactor was used to investigate the decomposition of toluene. Stainless steel (SS) external electrode (30 mm) was wrapped outside the external quartz tube with a 15 mm inner diameter. The inner SS electrode was placed inside the inner quartz tube having an outer diameter of 12 mm. The plasma was produced between the annular spaces of the coaxial quartz tubes. A variac was connected to the plasma generator to control the discharge power to the DBD reactor. In this study, the input power was varied from 5 to 40 W and measured using an energy meter. The length of the discharge zone depends upon the outer electrode (shortest electrode).^38^

The flow rate of the natural gas was controlled by using a computer-controlled mass flow controller, connected to the gas cylinder (BOC, UK). The natural gas was passed through a bubbler filled with toluene to add the vapours.

The gaseous products were measured by Varian 450-GC provided with a TCD (thermal conductivity detector) to monitor CH_4_ and H_2_, and FID (flame ionization detector) to detect lower hydrocarbons (LHC).

### 2.1 Definitions

The decomposition of toluene is defined as:
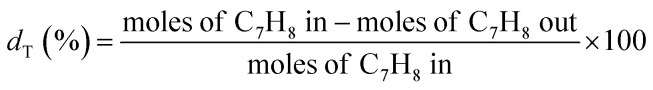


The yield of different product was defined as follows:
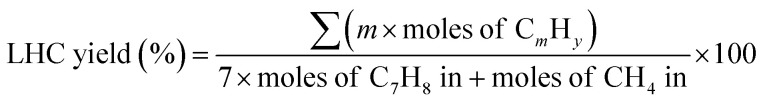
where *m* > 1
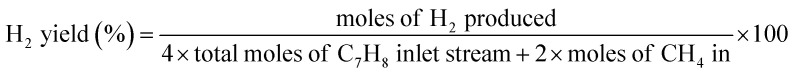

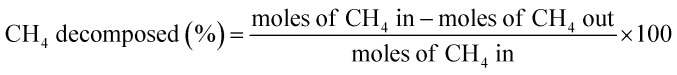


The energy efficiency and specific input energy (SIE) are calculated using the following formulas:^39^
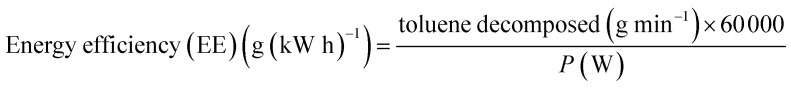

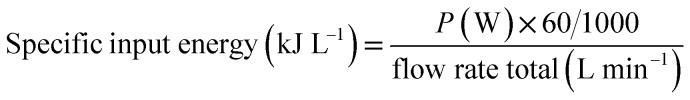


## 3. Results and discussion

### 3.1 Decomposition of VOC with changing the input power

**Fig. 2:**
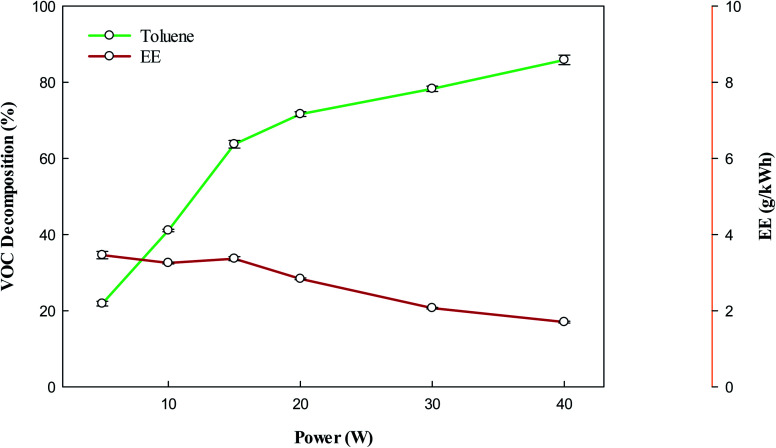
Effect of plasma input power on the conversion and energy efficiency of VOC. Reaction conditions: concentration, 33 g Nm^−3^; flow rate, 40 ml min^−1^; and temperature, ambient.

Fig. 2 shows the removal of toluene using the DBD reactor. It can be observed that the removal of toluene increases with increasing input power. It is well known that increasing plasma input power increases the number of high-energy electrons.^40,41^ These high-energy electrons are responsible to increase the decomposition of VOCs, whereas they can also produce reactive species upon collision with background gas,^42–44^ which can also contribute to the high conversion of VOCs. Therefore, at high power combined effect of energetic electrons and reactive species may contribute to enhance the decomposition of target compounds. The bond dissociation energy of the –CH_3_ group in toluene is 3.7 eV,^45^ which is lesser than the C–C bond between the methyl group and aromatic ring, and bonds present in the aromatic ring.^46^ Hence, the decomposition of toluene can proceed *via* the removal of H from the –CH_3_ group. The abstraction of H from CH_3_ form the benzyl radicals (eqn (1)), which can further react with reactive species/energetic electrons to produce lower hydrocarbons (eqn (7)). The reactive species have enough energy which can break the C–C bond between the aromatic ring and methyl group and produce phenyl radicals. These radicals have a tendency to agglomerate with benzyl radicals and produce undesirable solid residues (eqn (6)). However, there are enough highly energetic electron and reactive species which can rupture the aromatic ring directly and produce lower hydrocarbons. The decomposition of background gas (CH_4_) also produces CH_3_ and H radicals (eqn (3)). These reactive species may also contribute to decompose VOC (eqn (4) and (5)). Eqn (1)–(7) show some reactions involved during the decomposition of toluene:^39,47^

1 C_7_H_8_ + e → H + C_7_H_7_ + e

2 C_7_H_8_ + e → CH_3_ + C_6_H_5_ + e

3 CH_4_ + e → CH_3_ + H + e

4 C_7_H_8_ + H → H_2_ + C_7_H_7_

5 C_7_H_8_ + CH_3_ → CH_4_ + C_7_H_7_

6 C_6_H_5_ + C_7_H_7_ → polymerize

7 Toluene/intermediate products + e or active species → LHC (C_1_–C_6_) + e

### 3.2 Decomposition of CH_4_

**Fig. 3:**
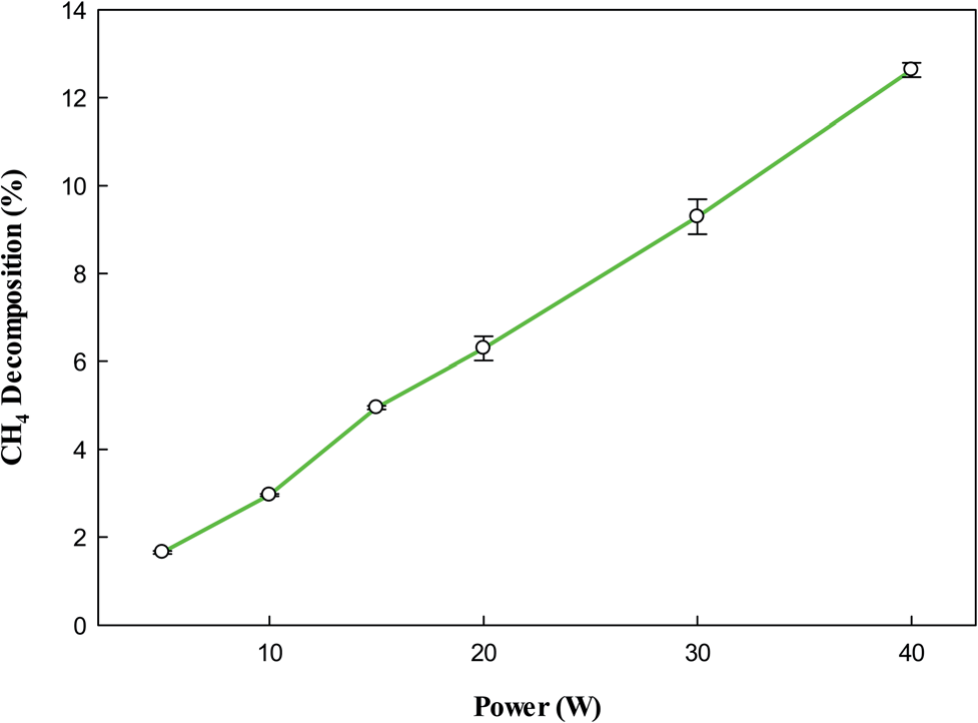
Effect of plasma input power on CH_4_ decomposition. Reaction conditions: concentration, 33 g Nm^−3^; flow rate, 40 ml min^−1^; and temperature, ambient.

Fig. 3 shows that the decomposition of CH_4_ also increases with increasing input power due to the presence of a high number of reactive species at higher input power, which play a vital role in NTP processes.^48^ However, the effect of power is the same, increases with increasing input power, due to abundant reactive species at high powers. The decomposition of CH_4_ into CH_3_ and H by NTP forms a variety of products, and the composition of these products heavily depends on the plasma input power.

### 3.3 Yield of gaseous products

**Fig. 4:**
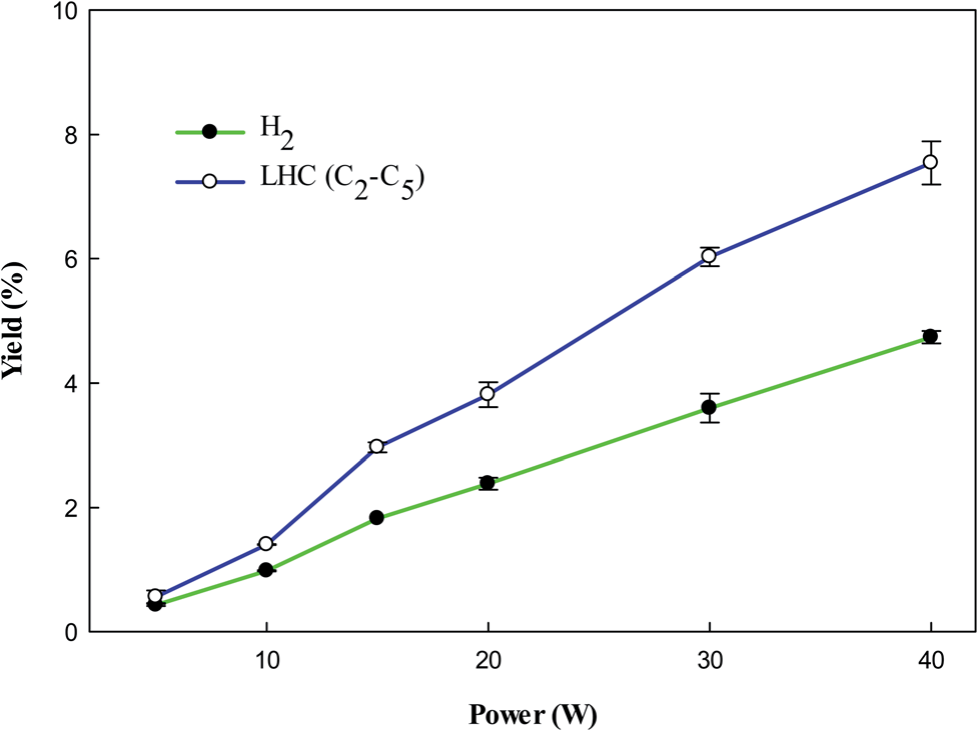
Effect of plasma input power on the yield of products. Reaction conditions: concentration, 33 g Nm^−3^; flow rate, 40 ml min^−1^; and temperature, ambient.

Fig. 4 shows the yield of gaseous products from toluene decomposition. It was observed that the yield of all products increases with increasing input power. At higher input power energetic electrons have enough energy which can rupture the aromatic ring resulting in the formation of LHC. Secondly, it can be seen (Fig. 4) that amount of H_2_ increases at higher power, which can also contribute to the formation of LHC. The decomposition of CH_4_ could also contribute to the production of H_2_ and LHC. Moreover, it was observed that the impact of energetic electrons generates different radicals such as CH_3_, CH_2_, CH, and H. These radicals combine to produce different LHC *via* reactions (8)–(13).^48–50^

8 CH_3_ + CH_3_ → C_2_H_6_

9 C_2_H_6_ + e → H + C_2_H_5_ + e

10 C_2_H_5_ + CH_3_ → C_3_H_8_

11 C_2_H_5_ + C_2_H_5_ → C_4_H_10_

12 C_3_H_8_ + e → H + C_3_H_7_ + e

13 C_3_H_7_ + C_2_H_5_ → C_5_H_12_

**Fig. 5:**
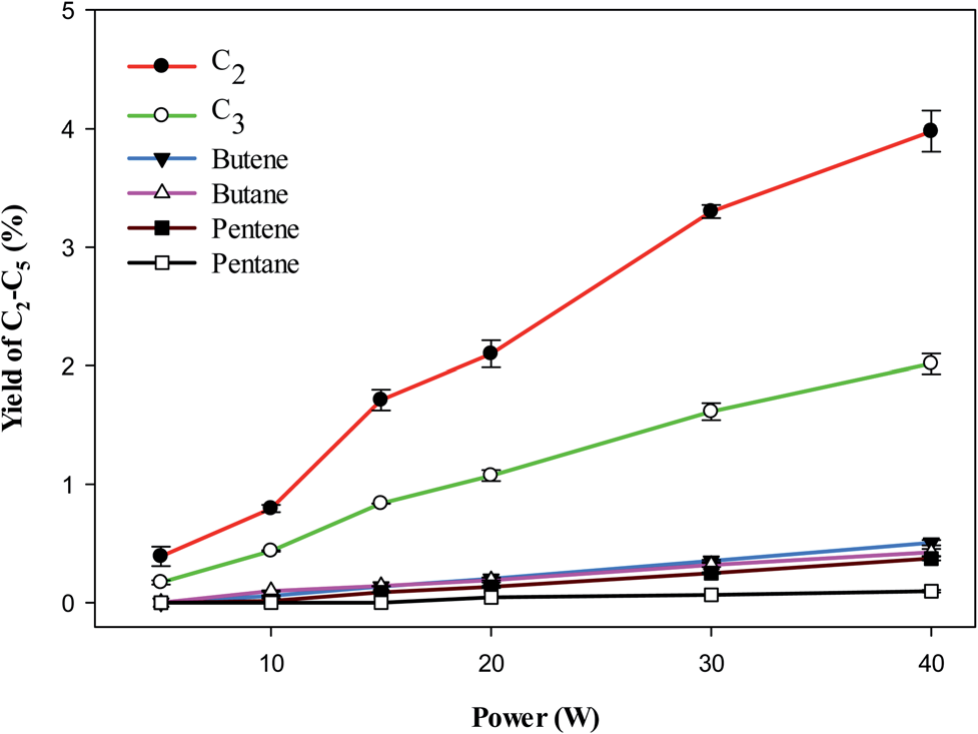
Effect of plasma input power on the yield of C_2_–C_5_. Reaction conditions: concentration, 33 g Nm^−3^; flow rate, 40 ml min^−1^; and temperature, ambient.

Fig. 5 shows the yield of individual LHC. The decomposition of VOC and background gas may contribute to the formation of LHC. In the plasma methane decomposition process the impact of electrons dissociate methane and produce CH_3_ (eqn (3)), which can combine to form C_2_ hydrocarbons (eqn (8)). Similarly, electron impact dissociation can produce a variety of radicals from different compounds (eqn (9) and (12)), which further recombine to produce >C_2_ hydrocarbons. Therefore, the yield of lower hydrocarbons and hydrogen collectively depends on the decomposition of VOC and methane carrier gas.

**Fig. 6:**
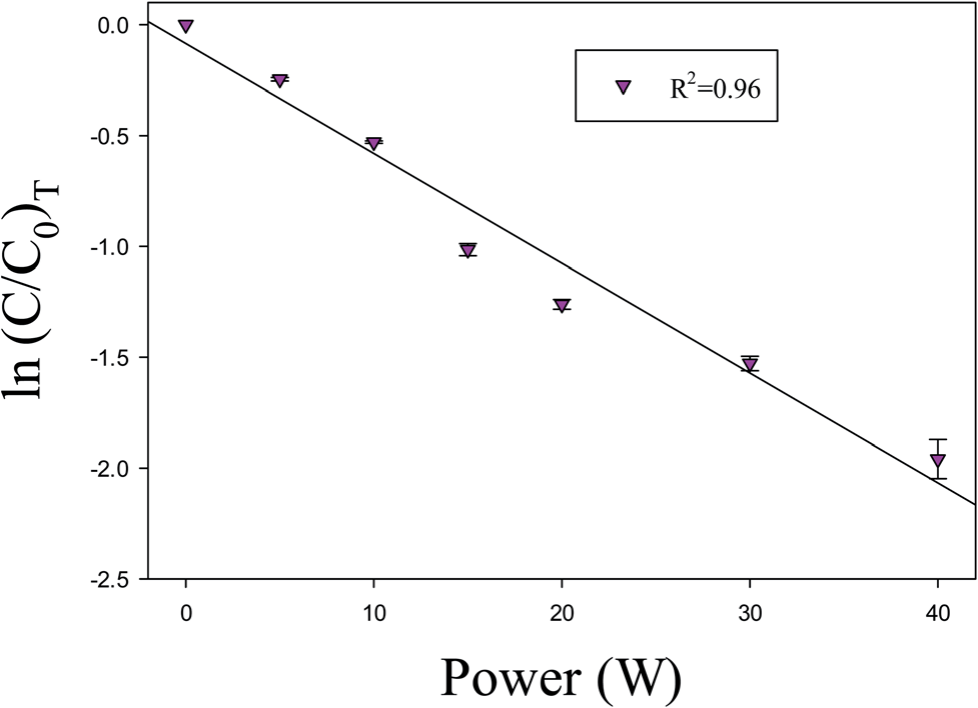
Power *versus* ln (*C*/*C*_0_). Reaction conditions: concentration, 33 g Nm^−3^; flow rate, 40 ml min^−1^; and temperature, ambient.

It has been observed that the reactions involved in NTP are power-dependent. The input power in the DBD reactor is the most important parameter. The input power plays a key role to produce active species, which produces ionization, dissociation, and excitation of background gas to start decomposition reactions. Therefore, it was reported that the reactions involved in NTP are energy-dependent.^51^Fig. 6 shows the variation of ln (*C*/*C*_0_)_T_ of toluene with input power. The value of *R*^2^ for linear regression is 0.96, which shows that the decomposition of VOC compounds in the current experiment is first ordered.

### 3.4 Effect of SIE

**Fig. 7:**
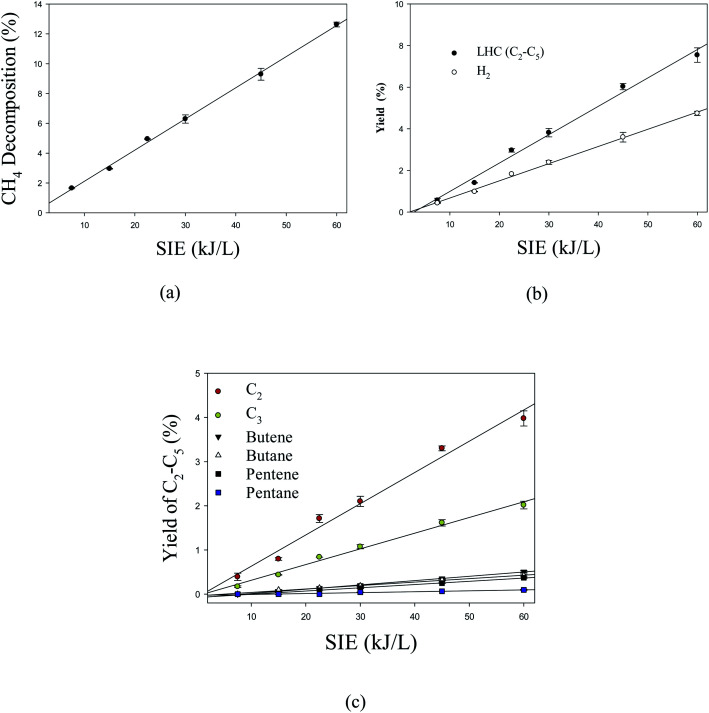
Effect of SIE on (a) the decomposition of CH_4_ (b) the product yield and (c) the yield of individual lower hydrocarbons. Reaction conditions: concentration, 33 g Nm^−3^; flow rate, 40 ml min^−1^; and temperature, ambient.

Fig. 7 shows the effect of specific input energy (SIE) on the decomposition of CH_4_ and products yield. It can be observed that the decomposition of methane and products yield increase linearly with increasing SIE. The SIE depends on input power and flow rate; however, the flow rate was kept constant in the current study. Due to this reason, SIE is closely related to plasma input power. Previous studies showed that increasing SIE enhanced the decomposition of methane and product yield.^52^ Overall, the major contributing factor is the input power because SIE only depends on power in this study.

## 4. Conclusions

The decomposition of toxic VOC (toluene) has been studied in CH_4_ gas, which is the major component of biogas using the DBD reactor. The results show that the removal of VOC could be achieved from CH_4_ gas by using NTP dielectric barrier discharged reactor. However, the removal of VOC depends on input power. The highest decomposition of toluene at 40 W and 2.86 s was 85.9%. The major gaseous products were H_2_ and LHC. The effect of input power was the same on the yield of the products as in the case of decomposition efficiency. The decomposition of background gas may be affected due to the formation of methane through the decomposition of toluene, which may occur due to the recombination of CH_3_ and H radicals. However, it is difficult to measure such an effect due to the presence of methane as the carrier gas. Hence, the NTP dielectric barrier discharged reactor could be employed to remove VOCs from the methane gas.

## Conflicts of interest

There are no conflicts to declare.

## Supplementary Material
